# First‐in‐Human Study of BAT4406F, an ADCC‐Enhanced Fully Humanized Anti‐CD20 Monoclonal Antibody in Patients With Neuromyelitis Optica Spectrum Disorders

**DOI:** 10.1111/cns.70126

**Published:** 2024-11-26

**Authors:** Hai Yu, Yuancheng Chen, Yunpeng Qi, Haijing Yang, Guoying Cao, Wenbo Yang, Size Li, Xiaolei Yang, Hai Wang, Jing Zhang, Xiangjun Chen

**Affiliations:** ^1^ Department of Neurology, Huashan Hospital, Fudan University and Institute of Neurology Fudan University Shanghai China; ^2^ National Center for Neurological Disorders Shanghai China; ^3^ Clinical Pharmacology Research Center, Huashan Hospital Fudan University Shanghai China; ^4^ National Clinical Research Center for Aging and Medicine, Huashan Hospital Fudan University Shanghai China; ^5^ Research Ward of Huashan Hospital Fudan University Shanghai China; ^6^ Bio‐Thera Solutions Ltd Guangzhou China

**Keywords:** BAT4406F, CD20, first‐in‐human, NMOSD, pharmacokinetics, safety

## Abstract

**Introduction:**

Neuromyelitis optica spectrum disorder (NMOSD) is a rare debilitating autoimmune disease of the central nervous system (CNS). This is the first‐in‐human dose‐escalation Phase I clinical study of BAT4406F, an antibody‐dependent cell‐mediated cytotoxicity (ADCC)‐enhanced fully humanized anti‐CD20 monoclonal antibody, in Chinese NMOSD patients.

**Patients and Methods:**

Using a “3 + 3” design and based on the planned algorithm of dose escalation, the enrolled NMOSD patients were sequentially assigned to one of the five dose‐escalation cohorts of BAT4406F with a single intravenous dose, and were then followed for a 6‐month observation period. The maximum tolerated dose (MTD) and dose‐limiting toxicity (DLT), safety, pharmacokinetics (PK), pharmacodynamics, and immunogenicity of BAT4406F were investigated, and the efficacy of BAT4406F in NMOSD was also preliminarily explored.

**Results:**

Fifteen Chinese NMOSD patients were enrolled to receive BAT4406F of escalated doses ranging from 20 to 750 mg. No subjects experienced DLT at the studied doses. BAT4406F injection exhibited favorable safety, with most of the adverse events (AE) of CTCAE Grade 1 or 2 in severity, and no Grade ≥ 3 adverse drug reactions (ADR) or serious adverse reactions occurred in any subjects. With the dose increase of BAT4406F, the maximum plasma concentration (*C*_max_), area under concentration‐time curve from 0 to the last measurable timepoint (AUC_0‐t_) and area under concentration‐time curve from 0 to infinity (AUC_0‐inf_) showed an increasing trend, whereas the mean clearance (CL_t_), terminal elimination rate (*λ*_Z_), and apparent volume of distribution (*V*_d_) decreased. The mean elimination half‐life (*T*_1/2_) was ranged from 9.0–16.4 days. PK profile of BAT4406F was generally nonlinear. BAT4406F led to a rapid and significant B‐cell depletion in all dose groups. Single administration of 500 mg or 750 mg maintains the CD19^+^ B lymphocyte count below 10/μL within the whole 6‐month observation period. Three subjects were antidrug antibody (ADA) positive and all of them were neutralizing antibody (NAb)‐negative. On day 99/180 postdose, several groups had decreased expanded disability status scale (EDSS) scores compared to baseline. During the observation period, NMOSD relapse occurred in two patients (13.3%) and the other 13 (86.7%) subjects remained relapse free.

**Conclusion:**

BAT4406F was well tolerated at doses up to 750 mg and showed an expected pharmacodynamic effect of significant and long‐term depletion of CD19^+^ B lymphocytes. It has also shown preliminary evidence of activity in NMOSD maintenance treatment, warranting further investigations.

**Trial Registration:**

ClinicalTrials.gov identifier: NCT04146285

## Graphical abstract

This is the first‐in‐human dose‐escalation Phase I study of BAT4406F, a fully humanized anti‐CD20 monoclonal antibody with enhanced ADCC effect. The safety, tolerability, pharmacokinetics (PK), pharmacodynamics, immunogenicity, and preliminary efficacy of BAT4406F injection on Chinese neuromyelitis optica spectrum disorder (NMOSD) patients were investigated.

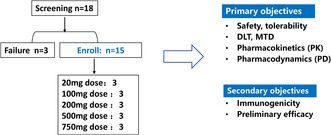

## 1 Introduction

Neuromyelitis optica spectrum disorder (NMOSD) is a rare immune‐mediated central nervous system (CNS) inflammatory demyelinating disease, primarily characterized by severe optic neuritis (ON) and longitudinally extensive transverse myelitis (LETM). It affects between 0.7 and 10 per 100,000 people, depending on geography and ethnicity, and is more common in women than men [1, 2, 3].

B‐cell involvement in NMOSD may include production of autoantibodies, T cell activation and cytokine production [4]. CD20 molecule is a non‐glycosylated phosphoprotein specifically labeled on the surface of B cells and is expressed on more than 95% of B cells. CD20 provides an important target for antibody‐mediated therapy in autoimmune diseases including NMOSD. Studies have shown that B‐cell depletion therapy by monoclonal antibody (mAb) against CD20 (such as rituximab) can reduce the recurrence of NMOSD and slow the progression of neurological dysfunction, and has a significant therapeutic effect. Rituximab has been included in treatment guidelines for NMOSD and recommended as first‐line treatment [5, 6].

BAT4406F is a glycosylation‐optimized fully anti‐CD20 human mAb of IgG1 subclass with enhanced antibody‐dependent cell‐mediated cytotoxicity (ADCC) effect. In an in vitro study using healthy human whole blood, BAT4406F showed significantly superior activity on depleting B cells compared to ofatumumab, ocrelizumab, and rituximab. After intravenous infusions of BAT4406F in cynomolgus monkeys, the CD20^+^ lymphocyte subgroups were below 0.5% at day 2 and the ratio was maintained until day 21; BAT4406F was found to effectively deplete B cells in a dose‐dependent manner, and has better B‐cell depletion activity than rituximab under the same dosage. Moreover, BAT4406F was shown to be well tolerated in animals.

In this first‐in‐human, dose‐escalation Phase I clinical study of BAT4406F injection in Chinese NMOSD patients, the overall objective is to assess the tolerability, safety, pharmacokinetics (PK), pharmacodynamics, and immunogenicity of BAT4406F in NMOSD patients. The efficacy of BAT4406F in NMOSD was also preliminarily explored.

## 2 Patients and Methods

### 2.1 Study Design

This was a Phase I, open‐label, dose‐escalation clinical study in NMOSD patients. This study was approved by the institutional review board and independent ethics committee of Huashan hospital of Fudan University, and was conducted in agreement with the Declaration of Helsinki and the Guidelines for Good Clinical Practice. Detailed informed consent was obtained from all screened patients. Eligible patients were enrolled in this trial and administered with a single intravenous infusion of BAT4406F followed by a 6‐month observation period.

The “3 + 3” dose‐escalation principle was adopted, and five fixed dose groups (i.e., 20, 100, 200, 500, 750) were set. The first three subjects were enrolled for each dose group, and the observation period for dose‐limiting toxicity (DLT) lasted for 28 days after administration. In the case where no DLT occurred as defined in the protocol, the patients who were assigned to the next higher dose level would receive treatment, until the MTD was found, or the study termination criteria were met. The primary endpoint was tolerability and safety of BAT4406F. Secondary endpoints included PK, pharmacodynamics, and immunogenicity of BAT4406F in NMOSD patients.

### 2.2 Patient Selection

Eligible patients are 18–65 years old, confirmed to have met the NMOSD diagnostic criteria developed by the 2015 International Panel for NMO Diagnosis (IPND) (can be with or without AQP4‐IgG) [1], had at least two relapses occurred within 2 years before screening or at least one relapse within 1 year before screening, had discontinued the immunosuppressive agents such as azathioprine, tacrolimus, mycophenolate mofetil, etc. within 28 days before the baseline. If the patients were on corticosteroids at screening, the dose should be 30 mg prednisone equivalent or below. Patients should have EDSS (Expanded Disability Status Scale) score ≤ 6 at screening. Patients were ineligible if they have been previously treated with anti‐CD20 mAb, have received intravenous immunoglobulin or plasma exchange within 1 month prior to screening, have received live vaccine within 4 weeks before baseline, or have a history of allergies to mAb or have severe allergic reaction to certain foods or drugs, or have abnormal liver function, kidney function or bone marrow reserve.

### 2.3 Treatment

BAT4406F was dissolved into 500 mL 0.9% sodium chloride injection and was administered intravenously. To reduce the risk of infusion‐related reactions, all the subjects received glucocorticoids and antihistamines at least 30 min before infusion. The initial infusion rate is 12 mL/h. In the absence of infusion reaction, the infusion rate is raised by 12 mL/h every 30 min to a maximum drop rate of 96 mL/h.

### 2.4 Pharmacokinetics

Blood samples were collected at predose, 5 min, 4 h and 12 h after the completion of infusion, and on days 2, 4, 8, 15, 29, 43, 71, 99 and 180 postdose, at each time point 2.5 mL blood was collected for PK analysis. Serum BAT4406F concentration was measured using a validated enzyme‐linked immunosorbent assay (ELISA). During the method validation, dilutional linearity, hook effect, selectivity, accuracy, precision, stability, specificity, parallelism and incurred sample reanalysis (ISR) were evaluated. The calibration curve range of the method was 40.0–2560 ng/mL, anchored points were 20.0 and 5120 ng/mL. The PK bioanalysis was performed by United‐Power Pharma Tech Co. Ltd.

### 2.5 Pharmacodynamics

About 3 mL of peripheral blood samples for B‐cell analysis were collected predose on day 1, and on days 4, 8, 15, 29, 43, 71, 99, 124, 152, and 180 after the treatment. The presence of BAT4406F in samples may interfere with CD20‐based detection of B cells, hence B‐cell surface marker CD19, which is co‐expressed with CD20 on B cells throughout most of their development, was used as a surrogate to monitor B cells [7]. CD19^+^ B lymphocyte ratio/count in peripheral blood at each follow‐up time point were detected by flow cytometry. The absolute number of CD19^+^ B cells was derived from the percentage of CD19^+^ cells and the absolute number of lymphocytes. B‐cell depletion was defined as a reduction in B‐cell counts by below 10/μL [8], and duration of depletion was defined as the interval between the drug treatment and last observations of > 10/μL in B‐cell counts in a subject. AQP4‐IgG serostatus was determined using ELISA on screening, predose on day 1, and on days 29, 43, 71, 99, 124, 152, and 180 postdose. Both the ELISA for AQP4‐IgG and flow cytometry for B‐cell analysis were performed at the laboratory facility at Huashan hospital of Fudan University.

### 2.6 Immunogenicity

Antidrug antibody (ADA) tests were performed at predose and on days 8, 15, 29, 43, 71, 99, and 180 postdose. At each time point about 5 mL of venous blood were collected for immunogenicity analysis. Anti‐BAT4406F antibody was determined on MSD platform by electrochemiluminescence immunoassay (ECLIA) and affinity capture elution (ACE) technique. In the case where the ADA test result was positive, neutralizing antibody (NAb) analysis was performed retrospectively. The detection of anti‐BAT4406F neutralizing antibody was based on a cytological test. The above bioanalysis was performed by United‐Power Pharma Tech Co. Ltd.

### 2.7 Study Measurements

DLTs were defined as Grades 3 or above drug‐related non‐hematological toxicity, and hematological toxicity such as Grade 4 neutropenia lasting 7 days or more, febrile neutropenia of Grade 3 or above, and Grade 4 thrombocytopenia, etc. Grade 3 or 4 infusion reactions are not DLTs.

Patients were evaluated for safety (vital signs, physical examination, clinical laboratory assessments, 12‐lead ECGs, infusion‐related reaction), tolerance, PK, pharmacodynamics (CD19^+^ B‐cell ratio/count), and immunogenicity (incidence of ADAs, their neutralizing potential and titer of positive). Adverse events (AEs) were graded with Common Terminology Criteria for Adverse Events (CTCAE) Version 5.0. The preliminary efficacy of BAT4406F was assessed by the time from day 1 to the onset of an NMOSD attack and the change from baseline of EDSS and MRI.

### 2.8 Statistical Analyses

The number and percentage of subjects who developed DLT were summarized by dose group. All AE summaries were presented by SOC and by PT as classified by the ICH Medical Dictionary for Regulatory Activities (MedDRA version 26.0). For laboratory tests (including hematology, urinalysis, blood chemistry, coagulation, AQP4‐IgG, etc.), 12‐lead ECG, and physical examination, the predose and postdose test results were cross tabulated. The CD19^+^B lymphocyte ratio/count at each time point and changes from baseline were statistically described. The anti‐BAT4406F ADA prevalence in serum was descriptively analyzed for each dose group. EDSS scores at Weeks 0, 12, and 24 were statistically described by dose group, respectively. The pre‐ and postdose MRI and ophthalmological findings were described by dose group using cross tabulation.

The PK parameters, including *C*_max_, time to maximum concentration (*T*_max_), terminal elimination half‐life (*T*_1/2_), clearance (CL), apparent volume of distribution (*V*_d_), mean residence time from time 0 to infinity (MRT_0‐inf_), area under the concentration‐time curve from time 0 to the last measurable timepoint (AUC_0‐t_) and from time 0 to infinity (AUC_0‐inf_), were calculated utilizing noncompartmental PK data analyses (PhoenixWinNonlin software, version 8.3, Certara, L. P., Princeton, NJ, USA). The statistics for PK parameters (arithmetic mean, geometric mean, standard deviation, coefficient of variation, maximum, median, minimum, and 95% CI, *T*_max_ expressed as median) were calculated and listed. Shapiro–Wilk test was used to test the normality of PK parameters. If the data were consistent with normal distribution, one‐way analysis of variance and Fisher's least significant difference was used to test the statistical significance of difference in PK parameters across dose groups. If data were not consistent with normal distribution, Kruskal–Wallis test was used. A difference was considered as statistically significant when *p* < 0.05. Power model was used to analyze the linearity between PK parameters and BAT4406F dose. All analyses were conducted using R (version 4.0.3) and SPSS software (version 26; IBM Co. Ltd., USA).

## 3 Results

### 3.1 Patients and Treatment

A total of 18 Chinese subjects were screened and 15 of them were enrolled. All the enrolled subjects have been carefully evaluated and confirmed to meet the NMOSD diagnostic criteria. Fourteen (93.3%) subjects were female and one (6.7%) subjects was male. All subjects are with Han nationality. The mean age ± SD was 42.13 ± 10.66 years old, ranging from 26 to 64 years old, of which 9 (60.0%) subjects were in the age group of 18–40 years old, and 6 (40.0%) subjects were in the age group of 41–65. All the patients had NMOSD relapse history. Thirteen subjects (86.7%) received previous NMOSD treatments including glucocorticoids and immunosuppressants. None of the subjects received prior non‐drug therapy for NMOSD.

**FIGURE 1:**
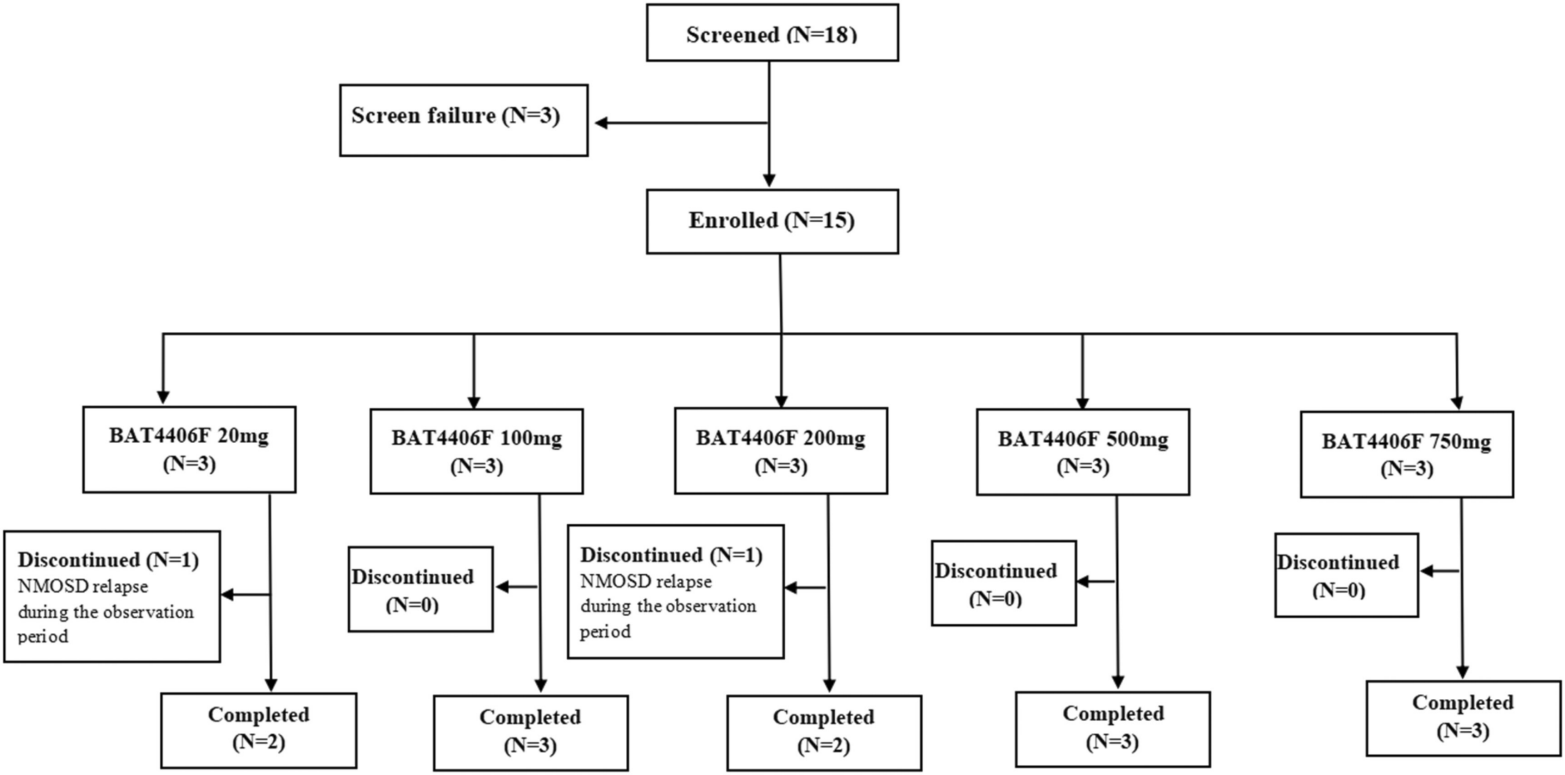
Subject Disposition.

Of the 15 subjects, every three subjects were enrolled in the each of 20, 100, 200, 500 and 750 mg groups, respectively. All 15 subjects (100%) were given BAT4406F injection according to the prescribed dose, and the dose compliance was 100%. Thirteen (86.7%) subjects remained relapse free and completed the study, whereas 2 (13.3%) subjects withdrew early due to disease relapse during the observation period (one subject from the 20 mg group and another one from the 200 mg group) (Figure 1).

### 3.2 Tolerability and Safety

After BAT4406F administration at the doses from 20 to 750 mg, all the 15 patients have completed the 28‐day DLT observation period and none developed DLT. The MTD for BAT4406F was not reached.

In terms of safety, a total of 120 AEs occurred in 14 (93.3%) subjects, including 56 adverse drug reactions (ADR) in 13 (86.7%) subjects. Most of the AEs were with the outcome of resolved, resolving, or stable. No Grade ≥ 3 ADRs, serious adverse reactions, AEs or ADRs leading to withdrawal occurred in any subject.

Most of the AEs were with CTCAE Grade 1 or 2 in severity. Only one AE of Grade 3 (PT: osteonecrosis) occurred in 1 subject from the 100 mg dose group, which met the criteria for serious adverse event (SAE). This subject had aseptic necrosis of head of femur diagnosed before screening. On day 70 after the drug treatment, the subject was hospitalized for hip replacement. The SAE was judged to be unlikely related to the investigational drug and was resolved. No Grades 4 or 5 AEs occurred.

Common ADRs with incidence > 10% (occurred in at least 2 subjects) by PT included: body temperature increased, heart rate increased, feeling cold, urinary tract infection, blood pressure increased, blood lactate dehydrogenase increased, fibrin D‐dimer increased, chest discomfort, fever, tachypnea, and allergic dermatitis, as shown in Table 1.

**TABLE 1:** Common ADRs with incidence > 10% (occurred in at least 2 subjects) by SOC and PT.

SOC/PT	20 mg group (*N* = 3)		100 mg group (*N* = 3)		200 mg group (*N* = 3)		500 mg group (*N* = 3)		750 mg group (*N* = 3)		Total (*N* = 15)	
	*E*	*n* (%)	*E*	*n* (%)	*E*	*n* (%)	*E*	*n* (%)	*E*	*n* (%)	*E*	*n* (%)
Total	18	3 (100)	8	3 (100)	9	2 (66.7)	8	2 (66.7)	13	3 (100)	56	13 (86.7)
Investigations	4	3 (100)	3	2 (66.7)	4	2 (66.7)	6	2 (66.7)	6	2 (66.7)	23	11 (73.3)
Body temperature increased	0	0 (0)	1	1 (33.3)	2	2 (66.7)	0	0 (0)	1	1 (33.3)	4	4 (26.7)
Blood pressure increased	0	0 (0)	0	0 (0)	0	0 (0)	2	1 (33.3)	1	1 (33.3)	3	2 (13.3)
Heart rate increased	0	0 (0)	0	0 (0)	0	0 (0)	1	1 (33.3)	2	2 (66.7)	3	3 (20.0)
Blood lactate dehydrogenase increased	0	0 (0)	0	0 (0)	0	0 (0)	1	1 (33.3)	1	1 (33.3)	2	2 (13.3)
Fibrin D‐dimer increased	1	1 (33.3)	0	0 (0)	0	0 (0)	1	1 (33.3)	0	0 (0)	2	2 (13.3)
General disorders and administration site conditions	2	1 (33.3)	2	2 (66.7)	2	2 (66.7)	0	0 (0)	4	3 (100)	10	8 (53.3)
Feeling cold	0	0 (0)	1	1 (33.3)	2	2 (66.7)	0	0 (0)	0	0 (0)	3	3 (20.0)
Chest discomfort	0	0 (0)	0	0 (0)	0	0 (0)	0	0 (0)	2	2 (66.7)	2	2 (13.3)
Fever	0	0 (0)	1	1 (33.3)	0	0 (0)	0	0 (0)	1	1 (33.3)	2	2 (13.3)
Infections and infestations	5	2 (66.7)	2	1 (33.3)	1	1 (33.3)	0	0 (0)	1	1 (33.3)	9	5 (33.3)
Urinary tract infection	3	1 (33.3)	1	1 (33.3)	1	1 (33.3)	0	0 (0)	0	0 (0)	5	3 (20.0)
Respiratory, thoracic and mediastinal disorders	1	1 (33.3)	0	0 (0)	1	1 (33.3)	1	1 (33.3)	2	1 (33.3)	5	4 (26.7)
Tachypnea	0	0 (0)	0	0 (0)	0	0 (0)	1	1 (33.3)	1	1 (33.3)	2	2 (13.3)
Skin and subcutaneous tissue disorders	0	0 (0)	1	1 (33.3)	1	1 (33.3)	1	1 (33.3)	0	0 (0)	3	3 (20.0)
Allergic dermatitis	0	0 (0)	0	0 (0)	1	1 (33.3)	1	1 (33.3)	0	0 (0)	2	2 (13.3)

Abbreviations: *E*, number of events; *n*, number of subjects.

Eleven AEs (they are all ADRs) occurred in 4 (26.7%) subjects were attributed to infusion reactions, all were of Grade 1 or 2, characterized as (by PT) fever, allergic dermatitis, chills, feeling cold, rash, dry throat, tachypnea, blood pressure increased, and body temperature increased (Table 2). The AE occurrence related to infusion reactions were comparable in the various dose groups.

**TABLE 2:** The occurrence of adverse events related to infusion reactions.

SOC/PT	20 mg group (*N* = 3)		100 mg group (*N* = 3)		200 mg group (*N* = 3)		500 mg group (*N* = 3)		750 mg group (*N* = 3)		Total (*N* = 15)	
	*E*	*n* (%)	*E*	*n* (%)	*E*	*n* (%)	*E*	*n* (%)	*E*	*n* (%)	*E*	*n* (%)
Total	0	0 (0)	2	1 (33.3)	4	1 (33.3)	3	1 (33.3)	2	1 (33.3)	11	4 (26.7)
General disorders and administration site conditions	0	0 (0)	1	1 (33.3)	1	1 (33.3)	0	0 (0)	2	1 (33.3)	4	3 (20.0)
Fever	0	0 (0)	1	1 (33.3)	0	0 (0)	0	0 (0)	1	1 (33.3)	2	2 (13.3)
Chills	0	0 (0)	0	0 (0)	0	0 (0)	0	0 (0)	1	1 (33.3)	1	1 (6.7)
Feeling cold	0	0 (0)	0	0 (0)	1	1 (33.3)	0	0 (0)	0	0 (0)	1	1 (6.7)
Skin and subcutaneous tissue disorders	0	0 (0)	1	1 (33.3)	1	1 (33.3)	1	1 (33.3)	0	0 (0)	3	3 (20.0)
Allergic dermatitis	0	0 (0)	0	0 (0)	1	1 (33.3)	1	1 (33.3)	0	0 (0)	2	2 (13.3)
Rash	0	0 (0)	1	1 (33.3)	0	0 (0)	0	0 (0)	0	0 (0)	1	1 (6.7)
Respiratory, thoracic and mediastinal disorders	0	0 (0)	0	0 (0)	1	1 (33.3)	1	1 (33.3)	0	0 (0)	2	2 (13.3)
Dry throat	0	0 (0)	0	0 (0)	1	1 (33.3)	0	0 (0)	0	0 (0)	1	1 (6.7)
Tachypnea	0	0 (0)	0	0 (0)	0	0 (0)	1	1 (33.3)	0	0 (0)	1	1 (6.7)
Investigations	0	0 (0)	0	0 (0)	1	1 (33.3)	1	1 (33.3)	0	0 (0)	2	2 (13.3)
Blood pressure increased	0	0 (0)	0	0 (0)	0	0 (0)	1	1 (33.3)	0	0 (0)	1	1 (6.7)
Body temperature increased	0	0 (0)	0	0 (0)	1	1 (33.3)	0	0 (0)	0	0 (0)	1	1 (6.7)

Abbreviations: *E*, number of events; *n*, number of subjects.

Abnormal and clinically significant results were observed in vital signs, physical examination, and ECG findings in a small number of subjects, which were related to subjects' medical history, concomitant medications, and/or related to the investigational drug.

Four subjects with normal ECG at baseline had clinically significant abnormal ECG after administration (including sinus bradycardia, counterclockwise transposition, frequent premature atrial contractions, sinus bradycardia, sinus arrhythmia, T‐wave changes), which were determined to be related to the subject's medical history or were adverse events (all were unlikely related with the study drug).

### 3.3 Pharmacokinetics

**FIGURE 2:**
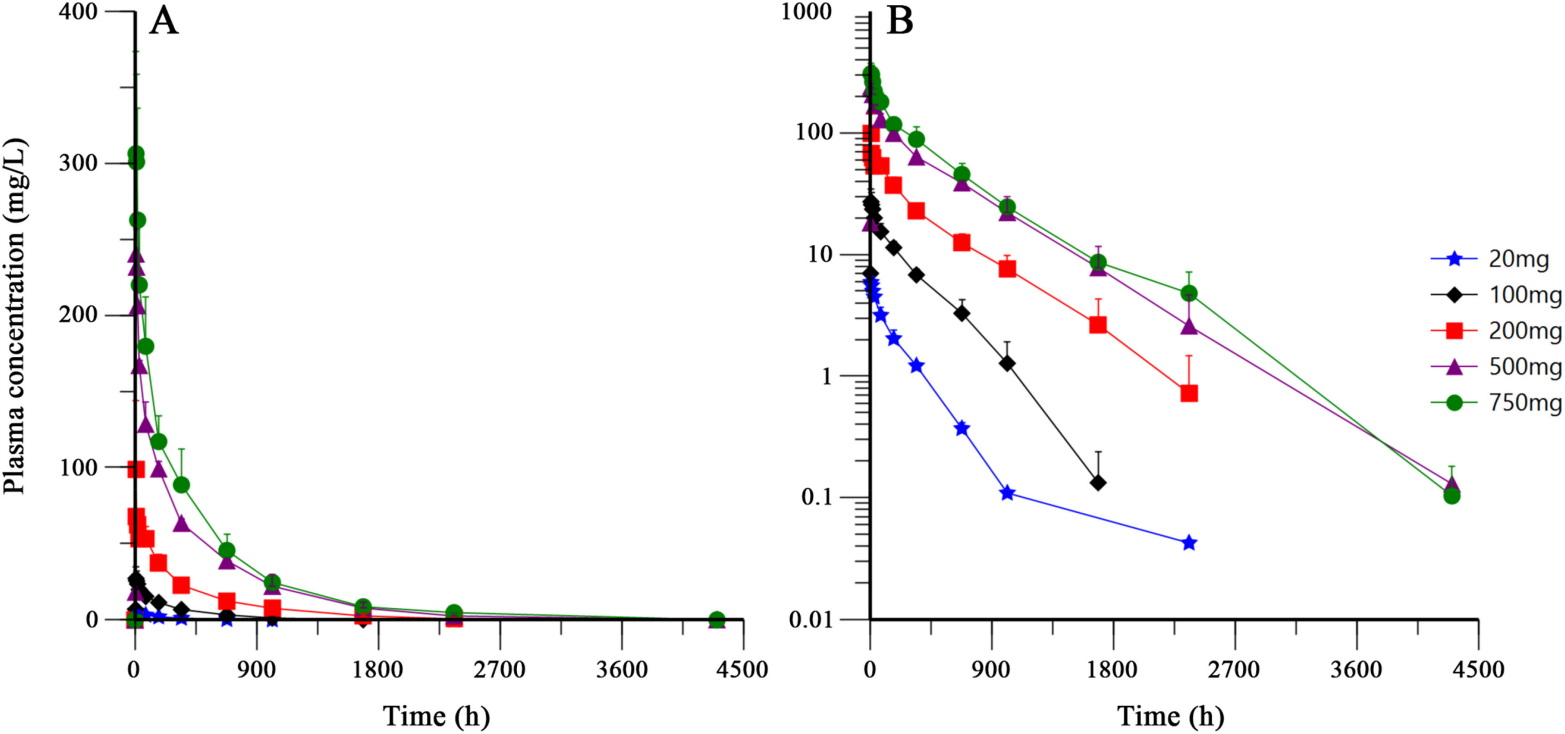
Plasma concentration–time curves following single infusion of 20–750 mg of BAT4406F (Mean ± SD, *n* = 3) (Left: Linear scale, Right: Semi‐Logarithm scale).

Figure 2 Shows the mean plasma concentration‐time profiles of BAT4406F after single administration. For all the dose groups, the elimination rate reduced obviously at about 100 h postdose, indicating that the drug distribution mainly occur from administration to 100 h postdose, whereas from 100 h to the end is mainly the elimination phase. There was a second turning point in the 20 mg dose curve (at about 900 h postdose). In the 100 and 200 mg groups, the elimination at the end of the curve seemed to be accelerating, suggesting a PK characteristics of the target‐mediated drug disposition (TMDD). In addition, most of the coefficient of variation of blood concentration at each time point was within 30%.

Pharmacokinetic parameters were shown in Table 3. With the dose increase of BAT4406F, the median *T*_max_ was in the range of 7.00 h to 10.90 h and generally tended to prolong with the dose increase. Similarly, *C*_max_, AUC_0‐t_ and AUC_0‐inf_ showed an increasing trend. The mean CL_t_, *λ*_Z_, and *V*_d_ decreased while the mean *T*_1/2_ prolonged. This suggested the slow elimination of BAT4406F.

**TABLE 3:** Pharmacokinetic parameters of BAT4406F (Mean ± SD, *n* = 3).

Dose (mg)	*C*_max_ (mg/L)	*T*_max_ (h)	AUC_0‐t_ (h·mg/L)	AUC_0‐inf_ (h·mg/L)	*λ*_Z_ (1/h)	*T*_1/2_ (h)	MRT_0‐inf_ (h)	CL_t_ (L/h)	*V*_d_ (L)
20	5.92 ± 0.76	7.00 (6.97, 11.10)	1190.44 ± 134.38	1240.21 ± 113.47	0.003 ± 0.001	238.81 ± 82.04	294.26 ± 77.1	0.016 ± 0.001	5.66 ± 2.29
100	27.17 ± 7.38	8.00 (7.58, 12.35)	7181.85 ± 1427.47	7225.58 ± 1467.11	0.003 ± 0	215.42 ± 25.36	337.51 ± 36.19	0.014 ± 0.003	4.35 ± 0.45
200	98.50 ± 45.54^a,b^	7.62 (7.58, 17.07)	25099.04 ± 5317.31	27629.4 ± 2454.67	0.002 ± 0^aa,bb^	375.88 ± 90.66^a,bb^	505.38 ± 116.85^aa,b^	0.007 ± 0.001^aa,bb^	3.90 ± 0.65
500	240.67 ± 26.58^aa,bb,cc^	11.25 (8.08, 13.98)	78831.99 ± 10819.97^aa,bb,c^	79344.72 ± 10415.14^aa,bb,c^	0.002 ± 0^a,b^	370.84 ± 27.66^a,bb^	542.73 ± 111.56^aa,b^	0.006 ± 0.001^aa,bb^	3.39 ± 0.30^a^
750	309.00 ± 63.02^aa,bb,cc,d^	10.90 (8.07, 12.30)	148191.18 ± 54337.92^aa,bb,cc,dd^	148252.33 ± 54368.28^aa,bb,cc,dd^	0.002 ± 0 ^a,b^	392.81 ± 35.38^aa,bb^	489.86 ± 93.08^aa,b^	0.006 ± 0.003^aa,bb^	3.16 ± 1.19^a^

*Note:* Compared to 20 mg group, ^a^ *p* < 0.05, ^aa^ *p* < 0.01; Compared to 100 mg group, ^b^ *p* < 0.05, ^bb^ *p* < 0.01; Compared to 200 mg group, ^c^ *p* < 0.05, ^cc^ *p* < 0.01; Compared to 500 mg group, ^d^ *p* < 0.05, ^dd^ *p* < 0.01, obtained from one‐way analysis of variance or Kruskal–Wallis test.

After being corrected for dose, the ranges of *C*_max_, AUC_0‐t_ and AUC_0‐inf_ were 0.27–0.49/L, 59.52–197.59 h/L and 62.01–197.67 h/L, respectively, also showed an increasing trend with the dose increase of BAT4406F. The Power model analysis revealed that the slope of AUC_0‐t_ and AUC_0‐inf_ and its 95% CI did not fall in the linear judgment interval, and the slope of *C*_max_ and its 95% CI only partially fell in the linear judgment interval, suggesting that the PK profile of BAT4406F was generally nonlinear at the doses ranging from 20 to 750 mg (data not shown).

### 3.4 Pharmacodynamics

**FIGURE 3:**
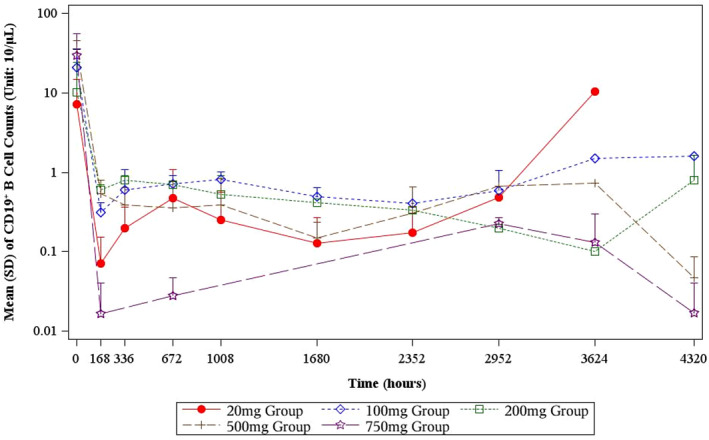
CD19^+^ B‐cell counts in each dose group (Mean ± SD, in Logarithm Scale).

After administered with BAT4406F, the CD19^+^ B lymphocyte decreased rapidly in all dose groups. Overall the higher is the dose level, the larger is the percent change from baseline. For example, at day 4 postdose, 500 and 750 mg BAT4406F deplete CD19^+^ B lymphocyte to a very low level (Table 4). At the following postdose time points, the CD19^+^ B lymphocyte counts continued to decrease. Single dose of BAT4406F administration maintains B lymphocyte at a low level, and the duration of B lymphocyte suppression and depletion depends on the dose. Specifically, for 20 mg group, the CD19^+^ B lymphocyte was firstly depleted and then started to raise from day 124, whereas the 100 mg group recovered from day 152, later than 20 mg group; 200 mg group had CD19^+^ B lymphocyte recovered at day 180. Single administration of 500 mg or 750 mg suppresses the CD19^+^ B lymphocyte count below 10/μL within the whole 6‐month observation period, and the 750 mg group had even lower postdose B lymphocyte level than the 500 mg group (Table 4, Figure 3).

**TABLE 4:** CD19^+^ B lymphocyte count in each dose group.

Visits	20 mg group	100 mg group	200 mg group	500 mg group	750 mg group
Baseline (Unit: 10/μL)					
*N* (Missing)	3 (0)	3 (0)	3 (0)	3 (0)	3 (0)
Mean ± SD	7.17 ± 7.56	20.98 ± 13.98	10.20 ± 13.86	36.29 ± 9.85	29.24 ± 25.88
Median	6.11	19.21	3.22	37.66	24.66
Day 4 (Unit: 10/μL)					
*N* (Missing)	3 (0)	3 (0)	3 (0)	3 (0)	3 (0)
Mean ± SD	1.25 ± 1.47	2.74 ± 3.89	2.86 ± 3.49	0.60 ± 0.26	0.07 ± 0.04
Median	0.87	0.71	1.03	0.75	0.08
Day 8 (Unit: 10/μL)					
*N* (Missing)	3 (0)	3 (0)	3 (0)	3 (0)	3 (0)
Mean ± SD	0.07 ± 0.08	0.33 ± 0.08	0.59 ± 0.07	0.54 ± 0.27	0.10 ± 0.14
Median	0.04	0.37	0.55	0.60	0.03
Day 15 (Unit: 10/μL)					
*N* (Missing)	3 (0)	3 (0)	3 (0)	3 (0)	3 (0)
Mean ± SD	0.20 ± 0.16	0.45 ± 0.42	0.75 ± 0.12	0.39 ± 0.25	0.08 ± 0.11
Median	0.23	0.26	0.73	0.51	0.04
Day 29 (Unit: 10/μL)					
*N* (Missing)	3 (0)	3 (0)	3 (0)	3 (0)	2 (1)
Mean ± SD	0.47 ± 0.61	0.71 ± 0.20	0.44 ± 0.26	0.36 ± 0.41	0.03 ± 0.02
Median	0.23	0.80	0.44	0.12	0.03
Day 43 (Unit: 10/μL)					
*N* (Missing)	2 (0)	3 (0)	2 (0)	3 (0)	1 (2)
Mean ± SD	0.25 ± 0.31	0.82 ± 0.19	0.52 ± 0.39	0.31 ± 0.35	0.00 ± NA
Median	0.25	0.81	0.52	0.15	0.00
Day 71 (Unit: 10/μL)					
*N* (Missing)	2 (0)	3 (0)	2 (0)	3 (0)	1 (2)
Mean ± SD	0.13 ± 0.14	0.50 ± 0.15	0.31 ± 0.15	0.15 ± 0.09	0.11 ± NA
Median	0.13	0.54	0.31	0.11	0.11
Day 99 (Unit: 10/μL)					
*N* (Missing)	2 (0)	3 (0)	2 (0)	3 (0)	2 (1)
Mean ± SD	0.17 ± 0.19	0.48 ± 0.23	0.39 ± 0.08	0.24 ± 0.27	0.04 ± 0.03
Median	0.17	0.59	0.39	0.11	0.04
Day 124 (Unit: 10/μL)					
*N* (Missing)	2 (0)	2 (1)	2 (0)	3 (0)	3 (0)
Mean ± SD	3.43 ± 4.17	0.59 ± 0.46	0.36 ± 0.23	0.26 ± 0.36	0.20 ± 0.05
Median	3.43	0.59	0.36	0.10	0.20
Day 152 (Unit: 10/μL)					
*N* (Missing)	2 (0)	2 (1)	2 (0)	2 (1)	3 (0)
Mean ± SD	5.38 ± 7.20	1.90 ± 0.54	0.18 ± 0.11	0.37 ± 0.52	0.12 ± 0.12
Median	5.38	1.90	0.18	0.37	0.09
Day 180 (Unit: 10/μL)					
*N* (Missing)	3 (0)	3 (0)	3 (0)	3 (0)	3 (0)
Mean ± SD	4.07 ± 6.35	4.25 ± 4.14	0.85 ± 0.61	0.13 ± 0.15	0.03 ± 0.02
Median	0.42	2.12	0.95	0.08	0.03

### 3.5 Efficacy

During the 6‐month observation period, 13 (86.7%) subjects remained relapse free, and 2 (13.3%) subjects relapsed at around day 40. Both of these relapsed subjects withdrew corticosteroids shortly after drug administration (at day 29 and day 4, respectively), so their disease recurrence may be related to the premature corticosteroid withdrawal, when the study drug has not displayed the efficacy; on the other hand, their respective doses, 20 mg and 200 mg, were probably still below the effective dose.

In the 20, 100, 200, 500, and 750 mg groups, on day 99 postdose, the mean change from the baseline in EDSS scores was 0.00, 0.00, 0.00, −0.17, and −1.00, respectively, where the 500 and 750 mg group had decreased EDSS from baseline. On day 180/early discontinuation, the mean change from baseline in EDSS score was 0.83, −1.00, 0.00, −1.25, and −0.50, respectively, where the 100, 500, and 750 mg group had decreased EDSS from baseline (Table S1).

The postdose MRI findings of optic nerve, brain and spinal cord in each dose group showed no obvious change from baseline; but in some subjects treated with doses at or above 100 mg, the MRI findings of spinal cord changed from abnormal and clinically significant to normal. Similarly, the ophthalmological findings changed from abnormal and clinically significant (related to the primary disease of NMOSD) to normal in some subjects in 200 mg to 750 mg groups. The above may be related to the therapeutic effect of the drug.

### 3.6 Immunogenicity

Three subjects were ADA positive (1 subject in the 20 mg group and 2 subjects in the 500 mg group), with an ADA prevalence of 20% (3/15) for this study. All of the three subjects were detected to be NAb negative. One subject in the 20 mg group and another one in the 500 mg group were predose ADA positive (pre‐existing antibody), with an predose antibody titer of 1:50 and 1:200, respectively; a third subject from the 500 mg group was with positive ADA only on day 8 (transient ADA), with an titer of 1:50 (Table S2). None of the subjects had treatment‐boosted ADAs. The PK, safety, and pharmacodynamics data of these 3 patients with positive ADA were generally similar to those with negative ADA in the same dose group, hence no obvious effects of ADA positivity on the above were observed.

## 4 Discussion

Although B‐cell depletion regimen has demonstrated its efficacy in reducing the NMOSD relapse risk, there is still a need for medical treatment options in NMOSD patients, especially for those without positive AQP4‐IgG. In this first‐in‐human Phase I study, the tolerability, safety, PK, pharmacodynamics, immunogenicity and preliminary efficacy of BAT4406F in Chinese NMOSD patients were evaluated.

All the 15 enrolled subjects completed the infusions and none of them met the DLT criteria as defined in the study. The recorded AE were found to be distributed evenly across all dose groups with no obvious evidence for a dose‐dependent effect. No Grade ≥ 3 ADRs, serious adverse reactions, AEs or ADRs leading to withdrawal occurred in any subjects. All the above showed that BAT4406F up to 750 mg was well tolerated in Chinese NMOSD patients of this study.

Monoclonal antibodies can cause infusion‐related reactions (IRR), most of which are caused by cytokine release [9]. Cytokine release induced by rituximab is dependent on the binding of rituximab to FcγR on immune cells rather than to targeted CD20 positive cells. These findings are consistent with the higher rate of IRR (in comparison to rituximab) with obinutuzumab, a glycoengineered anti‐CD20 mAb with increased binding to the FcγR IIIA (also called CD16) leading to increased ADCC and cytokine release [10, 11]. BAT4406F led to IRR in 4 (26.7%) subjects in this study, all were with the Grade 1 or 2 and had similar pattern to what has been observed in other anti‐CD20 monoclonal antibodies. These IRR of BAT4406F were then well resolved by short‐term cessation of the infusion, providing supportive care, and restarting the infusion at a slower rate [9, 12], implying that the IRR of BAT4406F is both expected and manageable.

With the dose increase of BAT4406F, the exposure increased and the *T*_1/2_ prolonged, whereas CL_t_, *λ*_Z_, and *V*_d_ decreased gradually. The power model analysis further indicated a nonlinear pharmacokinetics for BAT4406F. The above implied that BAT4406F showed a TMDD PK characteristics. This is the phenomenon in which a drug binds with high affinity to its pharmacological target site to such an extent that this affects its pharmacokinetic characteristics [13]. Due to its high affinity to the target, which is saturable because of the finite number of targets on the cell surface, this saturability causes the nonlinearity seen in TMDD models [14].

Most of the mAbs with B‐cell depletion activity have shown nonlinear PK characteristics. Bensalem reported that rituximab binds with target antigen irreversibly, and impact of underlying disease on PK could be explained using TMDD [15]. PK of rituximab in diffuse large B‐cell lymphoma was also nonlinear, where the clearance includes linear nonspecific elimination and time‐variable specific elimination, corresponding to the TMDD [16]. Yan et al. showed that PK of inebilizumab in NMOSD patients was nonlinear. The elimination was parallel first‐order and time‐variable nonlinear elimination, suggesting that this drug could be eliminated by receptor CD19 [17]. Gibiansky found that PK of CD20 mAb obinutuzumab is also nonlinear since the clearance reduced gradually along with time [18]. These findings are similar to what was observed in this study on PK of BAT4406F.

The enhanced ADCC effect of BAT4406F has translated into a superior B‐cell depletion activity than the marketed anti‐CD20 monoclonal antibodies in preclincal results. In this study, single dose of BAT4406F administration led to a significant and persistent B‐cell depletion, with the duration of B‐cell depletion in a dose‐dependent manner. Compared to the literature data of rituximab on B‐cell depletion in NMO and multiple sclerosis (MS) patients [19], BAT4406F showed stronger B‐cell depletion activity than rituximab in NMOSD patients. Increasing evidence shows that incomplete B‐cell depletion and/or B‐cell repopulation is associated with relapse risk in NMO [8, 20], the prompt and durable B‐cell depletion activity of BAT4406F as shown in this study can thus give pave to its promise in reducing disease recurrence. Moreover, it can be seen that one single dose of 500 mg or 750 mg BAT4406F could suppress the B‐cell count at a depletion state for 180 days, implying that the single dose regimen of BAT4406F may be considered for NMOSD maintenance treatment.

AQP4‐IgG is a highly specific serum autoantibody against AQP4 [2] detected in 60%–80% of NMO patients and is likely to be pathogenic [21, 22, 23]. In this study, subjects who have been evaluated to meet the NMOSD diagnosis criteria were enrolled, either with or without positive AQP4‐IgG at baseline. The two relapsed subjects were with positive baseline AQP4‐IgG. From the tolerability, safety, PK, pharmacodynamics, and immunogenicity data of this study, no obvious difference was observed between the subjects with or without positive baseline AQP4‐IgG.

BAT4406F displayed low immunogenicity in this study. For the two relapsed subjects, one subject from 20 mg group had positive ADA only at predose with the titer of 1:50, and the other relapsed subject was ADA negative, showing that ADA positivity may be unlikely related to NMOSD relapse. Moreover, the two subjects with positive predose ADA had no previous use of any anti‐CD20 antibodies or other biologic agents, leaving the underlying reasons for their pre‐existing positive ADA to be further investigated. A third subject with positive ADA had positive ADA only on day 8, with a titer of 1:50. This is considered to be transient ADA and is assumed to be with no clinical significance.

In this study, BAT4406F has preliminarily shown its efficacy in reducing the NMOSD relapse risk, and it was safe and well tolerated across the studied dose range. The observed clinical activity is encouraging; however, this study has some limitations. Firstly, this is an open‐label study without a control arm or a historical control, hence the obtained results may be biased and difficult to interpret. Secondly, due to the small number of the subjects, there may exist the inter‐individual variability in the observed pharmacokinetic parameters, and more safety signal are to be uncovered to better characterize the safety profile of BAT4406F.

## 5 Conclusion

In this study, BAT4406F exhibited favorable safety and tolerability in Chinese NMOSD patients, and no subjects developed DLT in the studied dose range. BAT4406F showed a nonlinear pharmacokinetics, exhibited a significant B‐cell depletion effect with the duration of B‐cell depletion in a dose‐dependent manner, and displayed a relatively low immunogenicity. After treatment with BAT4406F, most subjects remained relapse free, showing that BAT4406F has the potential to reduce the recurrences of NMOSD. Further elucidation of the efficacy and safety profile of BAT4406F in NMOSD is recommended.

## Author Contributions

X.C. and J.Z. designed the framework of manuscript, provided views and revised the manuscript; X.C., H.Y., and W.Y. conducted the study at the clinical site; J.Z., Y.C., H.Y., G.C., and S.L. analyzed the pharmacokinetic data; H.Y., Y.C., and Y.Q. prepared the manuscript; Y.Q., X.Y., and H.W. managed the study; all authors revised and approved the final manuscript.

## Consent

Written informed consent was obtained from all participants.

## Conflicts of Interest

The authors declare no conflicts of interest.

## Supporting information


Tables S1–S2.

## Data Availability

The data that support the findings of this study are available from the corresponding author upon reasonable request.
