# Supplementary material for: First‐in‐Human Study of BAT4406F, an ADCC‐Enhanced Fully Humanized Anti‐CD20 Monoclonal Antibody in Patients With Neuromyelitis Optica Spectrum Disorders
Source: CNS Neurosci Ther. 2024 Nov 26;30(11):e70126. doi: 10.1111/cns.70126 (PMC11598743; doi:10.1111/cns.70126)
Supplement: Supplementary file 1 — Tables S1–S2. [file CNS-30-e70126-s001.docx]

**Supplementary material**

**Table. S1 EDSS Scores of Subjects in Each Dose Group**

**Table. S2 Subjects with Positive Antidrug Antibody and Their Titers**

| **Dose Group** | **Subject ID** | **Time Points with Positive ADA** | **ADA Titer** | **Neutralizing antibody** |
| --- | --- | --- | --- | --- |
| 20 mg | 01R001 | Predose | 1:50 | Negtive |
| 500 mg | 01R010 | Day 8 | 1:50 | Negtive |
| 500 mg | 01R011 | Predose | 1:200 | Negtive |
|  |  | Day 8 | 1:400 | Negtive |
|  |  | Day 15 | 1:200 | Negtive |
|  |  | Day 29 | 1:100 | Negtive |
|  |  | Day 43 | 1:400 | Negtive |
|  |  | Day 71 | 1:100 | Negtive |
|  |  | Day 99 | 1:50 | Negtive |
